# Transition in Residues: On Depleted Oil Wells, Radioactive Geophysics, and the Origins of the Twentieth Century's Energy Mix

**DOI:** 10.1002/bewi.202400019

**Published:** 2025-06-16

**Authors:** Michiel Bron

**Affiliations:** ^1^ Faculty of Arts and Social Sciences Maastricht University 6200 MD Maastricht Netherlands

**Keywords:** applied geophysics, energy transition, geophysics, nuclear energy, oil, radioactivity

## Abstract

The oil and uranium industries always have been intertwined. Both industries are inherently global and span an extensive geological history. The formation of uranium and oil deposits, and their eventual extraction, is a story circling through early planetary history, continuing in depleted oil wells in Germany, Canada, and France, and lingering well into the second half of the past century. Understanding this history proved to be the key for two businesses that would shape the later twentieth century: the oil and nuclear industries. Oil companies are among the very first to integrate new quantum mechanics and knowledge about radioactive decay into their search for oil. This article locates the origins of this interconnectedness in the emergence of applied geophysics. Based on case studies to the experiments and research projects of geophysicist Richard Ambronn and the studies by the oil service company Schlumberger into measuring radioactive decay as a method of determining underground sediments and finding oil during the 1920s and 1930s, this article argues that the depleted oil sources at Pechelbronn and Celle formed the basis of both industrial and academic developments in the knowledge of radioactivity, geophysics, and petroleum.

## 1 The Oil Memories of Lower Saxony

In the German region of Lower Saxony, between the cities of Hannover and Celle, memories of the local oil extraction are still alive. The German oil museum in the nearby town of Wietze reminds visitors of the oil drilling that marked the area during the first decades of the 20th century. Since the first borehole was drilled to extract the crude oil from the rich, local tar sands in 1858, wildcatters and industrial parties searched for the resource and built an industry that would shape the history, memories, and landscapes of the region. Especially during the first decades of the 20th century, the Lower Saxony region was booming. More than 2000 wells were drilled until the early 1930s, extracting at least seven different varieties of oil. In these early years, with a high point between 1910 and 1920 before the production started to decline, several dozens of oil companies supplied about eighty percent of the German oil production at the time.^1^

In addition to the entrepreneurs that hoped to share in the oil wealth, the wells also attracted another audience: scientists who wanted to take advantage of the opportunities this new industry offered for their geophysical research. Even today, the intertwined history of industry and early geophysics is kept alive with business leaderships in geophysics at the Celle University of Applied Sciences and with companies like Mobile Geophysical Technologies Gesellschaft mit beschränkter Haftung and GeoEnergy Celle eingetragener verein (e.V.) operating from the region.^2^ Also, the German Oil Museum's collection reminds visitors that building the oil industry functioned as an incubator for geophysical innovations. The exhibitions show geophysical measuring instruments like the rotary balance and gravimeters that recall the engineering developments that were developed within the local oil industry, made possible by new developments of geophysical knowledge by the scientists.^3^

In this place a transition originated that propelled oil from a niche product, mainly used for greases and medicaments, to a primary energy source in the worldwide energy mix during the twentieth century, and started the development leading toward the addition of nuclear energy. In this article, I combine the fields of history of science, business history, and transition studies to show how the entanglement between early geophysicists and local developments in oil fields such as the wells in Lower Saxony formed the basis for the subfield of applied geophysics and the development of geophysical knowledge about natural radioactivity with far‐reaching consequences for developments in both the later oil and nuclear industries. The region of Lower Saxony provides a good example of places where oil infrastructures were used for conducting the applied research took place that would introduce technologies to measure radioactivity. Especially the research by the German geophysicist Richard Ambronn, conducted at the boreholes at Celle, proved crucial to the developments of the later oil and uranium exploration industries.

In the historiography of science, multiple scholars have studied the emergence of academic geophysics in the context of growing industrial interests.^4^ Aitor Anduaga has shown how the American oil industry affected the epistemic foundations of the academic discipline of crustal seismology, while Geoffrey Bowker and Douglas Yates in their studies on the French oil service company Schlumberger have argued that the development of applications of geophysical methods for electric well logging techniques was heavily influenced by the spillovers between academic geophysics and the early oil industry. Schlumberger was a service company that developed technologies to explore for oil, and became, according to Bowker, an important incubator for new geophysical technologies and knowledge in the oil industry such as the electric well logging device.^5^ Also historian of the earth sciences, Paul Lucier, has extensively argued that the formation of the earth sciences went hand in hand with industrial interests in extraction of raw materials.^6^

An understudied part of the history of industrial oil exploration and early geophysics, however, is the development of the knowledge about radioactivity and the early quantum mechanics‐based geophysical technologies such as radioactive well logging. This development, that took place within the research of individual geophysicists like Richard Ambronn and in the production fields of geophysical companies such as the French company Schlumberger, provides an essential link between the historiography of early atomic physics and the later industrial applications of nuclear fission in the United States Manhattan Project and the many subsequent attempts to establish nuclear industries in other countries. Within these developments, the oil industry played a major role. Already in the Manhattan Project various oil companies and oil service firms were contracted to search for uranium, built and manage production plants and conduct geochemical research. After the Second World War, oil actors continued their involvement with uranium mining projects, culminating in a ubiquitous oil involvement in the nuclear industry during the 1970s.^7^

Recently, historians within the subfields of science diplomacy and the history of science have shown how oil geophysicists and geophysical methods derived from the oil industry played an important role in establishing the uranium industry and the spillovers between the oil and nuclear industries *after* the Second World War.^8^ This article shows, however, that the basis for this involvement was grounded in the knowledge of radioactivity and atomic physics gained between 1896 and 1940 in many studies conducted on boreholes and oil field waters, and connects the geoscientific spillovers between both industries to the overarching framework of the an energy transition. The German geophysicist Richard Ambronn used the boreholes in Celle for his geophysical research and used his studies to propose the establishment of the subfield of “applied geophysics” in which he paid extensive attention to his studies on radioactivity and its geophysical applications. This article examines this effort by Ambronn in a broader context of several geophysicists in North America, Western Europe, and the Soviet Union, all of whom used oil fields for their research on the radioactive properties of minerals. This knowledge led to both new knowledge about radioactive mineral deposits and new techniques for finding oil. Techniques that would be commercialized via the laboratories of industrial oil firms such as Schlumberger, and later Royal Dutch Shell, Lane Wells Company and Gulf Oil. These technologies would then provide the entry point for the oil industry to enter the nuclear age.

By focusing on the early connections between the oil industry and geophysical research on radioactivity, this article retells the traditional story of an energy transition. Where more conventional stories on the emergence of nuclear energy in relation to the oil industry tend to focus on the various transition pathways incumbent oil actors embarked on when faced with the rapid post‐war developments of the nuclear industry, this article highlights the already established links between early atomic physics and a growing, but definitely not yet dominating, oil industry.^9^ By focusing on these early entanglements, and positioning them in oil sites such as the fields in Lower Saxony, this article shows how the later energy transitions was partly founded on early, pre‐Second World War spillovers in the emerging subdiscipline of applied geophysics.^10^

Based on new research to the publications of geoscientists as Richard Ambronn and his predecessors in journals such as *The London, Edinburg and Dublin Philosophical Magazine and Journal of Science*, and archival sources on early techniques to measure radioactivity at the Schlumberger archives, this article shows that the specific qualities of sites such as the oil wells in Lower Saxony—sites with oil wells which seemed to be depleted—proved to be crucial to this development. The sites where the production started to decline after 1920s offered opportunities to repurpose the oil infrastructures to training grounds for geophysicists and experimental geophysical technologies, showing how the origins for the transitions toward the energy mix of the later twentieth century were constructed in these literal residues of the oil industry.

## 2 Applied Geophysics

*Methoden der Angewandten Geophysik* in 1926 (translated in 1928 into English by the American petroleum geologist Margareth C. Cobb as *Elements of Geophysics as Applied to Explorations for Minerals, Oil and Gas*) on the applications of geophysical methods to mineral and oil and gas exploration by Richard Ambronn proved a turning point in the history of radioactive measurements in the oil industry. Ambronn, born in May 1887 in Hamburg, was a German geophysicist. After studying physics, he earned his doctorate in 1912 with physicist Woldemar Voigt at Georg August University of Göttingen on the electrical conductivity of crystals. Before Ambronn would join the NSDAP in 1932 as chairman for the local faction of the nazi party in Göttingen, he spent two decades doing geophysical research, particularly in the Celle oil drilling boreholes.^11^ There he investigated the properties of cores taken from the oil well. By doing so, he was especially focused on measurements of radioactivity—an interest which was sparked by earlier research conducted by Stefan Meyer and Egon Schweidler, Marie Curie, Ernest Rutherford and Frederick Soddy, and Hans Wilhelm Geiger and Walter Mackower—but also worked on magnetic prospecting methods and measurements of the distribution of heath underground.^12^

These studies culminated in the, well received, publication of *Methoden der Angewandten Geophysik*.^13^ With his book, Ambronn aimed to contribute to the growing attention to the systematic use of geophysical methods of exploration in mining and hydraulics, in the solution for water supply problems, and as an auxiliary to geologic research. According to Ambronn, the publications that had already reported on many individual problems or had contributed to limited parts of the field focusing on the applications of geophysics were scattered throughout the periodicals of the whole world. With his book, however, Ambronn hoped to bring together these different research projects in a new subfield of applied geophysics. Although this subfield would never obtain a completely independent position since it would always be supportive to practical geologists and industrial interests, Ambronn claimed that the geologist and physicist were indispensable to one another in the planning and execution of geophysical investigations. By doing so, Ambronn hoped to sell the geophysical research as a valuable asset to the oil industry (a goal he would succeed in as this article will later show).^14^

To make this point, the book presented all kinds of different methods for geophysical research that showed practical applications for finding minerals, vaults, and sediments. Among these methods, the use of radioactive measurements played a significant role. Following the dissociation hypothesis of Ernest Rutherford and Frederick Soddy, Ambronn stated that over time radioactive derivatives developed from a radioactive base.^15^ This dissociation was accompanied by the emission of different kinds of rays—most notably alpha, beta, and gamma rays—carrying “extraordinarily” amounts of energy. These rays could be used for the detection and classification of the material concerned, argued Ambronn. By measuring the emitted rays and comparing the results with the half value period of each initial substance of radioactive decay series, uranium, and thorium, an estimation could be made on the geological content of the sediments.^16^

By far the most important method of measuring radioactivity was the measurement of ionization in gases excited by the rays of radioactive materials. When gases are permeated by alpha, beta, or gamma‐rays, positive and negative ions are produced, splitting up from the neutral gas molecules. By creating an electric field, the two kinds of ions start to move in opposite directions toward the electrodes that are producing the field, enabling the saturation of the electrical current. The strength of the saturated current, appreciated for its theoretical simplicity, can then be used for identifying the radioactive element by measuring the power of the penetration. For example, because beta‐rays are characterized by their low‐penetrating power, this allows for measurements with different absorbing materials to calculate the individual components of the ray and locating the amount of the different decompositions of radium in the material.^17^

For a novice in early quantum mechanics this is a seemingly complex procedure, but Ambronn argued, based on previous research, that this technique opened ways to locate sedimentary layers, even in situ, without removing the sample from the soil. In his book, Ambronn proposed several methods of lowering boxes containing an apparatus suitable for course measurements into boreholes. In the boreholes, gas‐filled enclosures in the boxes would be penetrated by different types of rays, making clear which radioactive elements were grouped together at different heights underground. In this way, Ambronn offered a practical application for the research he had conducted himself in the muddy boreholes of Celle's oil drilling sites. Not only was it possible to study radioactivity and classify radioactive minerals, but this type of research would actually allow geophysicists to locate specific sedimentary layers where radioactive minerals tend or do not tend to deposit, making it easier to locate vaults, deposits, and reservoirs underground.^18^

## 3 Radioactive Geophysics in the Residues of the Early Oil Industry

For his studies Ambronn depended on the earlier work by geologists, atomic physicists, and early geophysicists. His theories were building on the fundamental research conducted by many of the leading physicists such as Henri Becquerel, Marie Curie, Ernest Rutherford and Frederick Soddy, Hans Wilhelm Geiger, and Makower but also on the studies by the Irish geologist John Joly.^19^ Especially the work by Joly on radioactivity and geology proved fundamental for Ambronn's claims on measuring natural radioactivity. During the first years of the twentieth century, Joly conducted research to quantities of radium and thorium in a wide range of materials, ranging from rocks and minerals to sea water. These studies culminated in the 1909 publication of a, now‐famous work called *Radioactivity and Geology*. In this book he argued that the radioactive element was present, in varying concentrations, all over the earth.^20^

The studies by Joly served as a source of inspiration for a variety of geologists and early geophysicists wanting to study the presence of radioactivity in nature and measure the radiation emitted by various kinds of rocks, fluids, and gases. By conducting a series of tests along borings, tunnels, and galleries, scientists documented varieties of radioactivity in multiple kinds of sedimentary layers, although without being able to establish consistent relations with geologic formations.^21^

To conduct their research, the geologists often made use of already established infrastructures that had uncovered sedimentary layers that otherwise would have been too expensive to reach for fundamental research such as tunnels, mining shafts, and boreholes. In the Austrian Alpes, physicists Heinrich Mache and Max Bamberger studied the Tauern tunnel, while in Switzerland John Hewett Jellet Poole investigated the Lötschberg tunnel.^22^ On the South American continent, Fletcher studied the sedimentary layers in tunnels in the Andes.^23^ Also, mining projects were used. For example, the British geophysicist Wiliam Frederick Smeeth found in the Kolar gold mine in Karnataka, India, a constant radium content up to a depth of thousand meter.^24^

Many of these studies took place in the infrastructures of the rapidly developing oil industry. Physicists such as Arthur Stewart Eve, Douglas McIntosh, and Eli Franklin Burton made use of the infrastructure provided by the early oil industry to study oil wells in the United States and Canada.^25^ In the Soviet Union, the famous geochemist Vladimir Vernadsky studied the oil field waters at the Ukhta oil field in the Komi Republic.^26^ Also in Western Europe, oil fields regularly attracted physicists and geologists wanting to study the radioactivity of oil field waters, petroleum, and underground minerals. Like Ambronn conducting his research on the oil boreholes near Celle, the German geochemist Ernst Hendrik Büchner used the boreholes drilled by the oil industry in a well at Baarlo in Holland‐Limburg.^27^

Although the various examples spanned half the globe, there was one element that tied these oil wells together. Many of the wells had already been drilled during the first decades of the industrial search for oil that had started with the drilling in Ontario, Canada, and in Lower Saxony in 1858. Often these sources had generated great prosperity to the first oil entrepreneurs, or subsequent industrial parties, and production from these sources had brought a large‐scale infrastructure that made it more feasible to reach the oil wells and extract the oil. After multiple decades of extracting oil on an industrial scale however, many of these oil wells seemed to have reached their peak production during the first decades of the 20th century. In the case of water‐drive reservoirs, oil wells often produce at a near constant rate until the encroaching water reaches the well. This causes a sudden decline in oil production reflected in the extraction of water and mud instead of oil.^28^

However, where the industrial interests in the depleting oil wells started to shift away to more productive, newer oil fields, the depletion also offered new opportunities to repurpose the existing oil fields into training grounds for early geophysicists. In an ever competitive context, oil firms from the start of the twentieth century onward increasingly looked for new applications of academic geophysics and chemistry to improve their production rates and keep ahead of other companies.^29^ Using the already established infrastructure at oil fields with declining production rates to train geophysicists and new geophysical methods therefore benefitted the industry both by offering access to new developments and extending the utility of the investments already made for the infrastructure around the boreholes. Historian Geoffrey Bowker shows how the competitive edge in electrical well logging of the French Schlumberger company was founded on the access to the local oil well at Pechelbronn, in the Alsace region of France. This oil field was already nearly depleted which made it expensive to drill for oil and there was an urgent need for new, innovative methods to reduce costs. Pechelbronn was then repurposed by Schlumberger to serve as a site for the apprenticeship for new geophysical engineers of the company, offering the opportunity for independent entrepreneurs and scientists to conduct their experiments. In this way, Schlumberger used Pechelbronn to analyze, meet, and possibly sabotage or recruit other geoscientists trying to research ways of finding oil and other minerals.^30^

For studies into the measurement of radioactivity in particular, the inherent quality of the boreholes being almost depleted proved crucial. In the muddy boreholes, polluted with underground water, measuring radioactive decay by lowering gas‐filled enclosures in the boreholes delivered more, and often better, results than comparable techniques like electric well logging. Due to that radioactivity is not affected by the nature of the mud, while many contemporary electric well logging devices quickly became useless in salt‐ and water‐saturated mud, logging based on radioactivity could still produce results in the muddy boreholes that were used to train the oil geophysicists.^31^ Sometimes this even meant that experiments with measuring radioactivity showed that some oil reservoirs thought to be almost depleted still contained unextracted oil reserves, or new fields were discovered in places previously thought not to contain any oil.^32^

Also, Ambronn, in his *Methoden der Angewandten Geophysik*, emphasized how varying geological circumstances influenced which geophysical methods should be applied. Especially the sites around Hannover, where Ambronn conducted his research, provided difficulties in making assessments with only electric well logging techniques. To solely depend on these techniques would not result in decisive answers on the presence of oil reservoirs according to Ambronn. Other methods, more adjusted to measure the magnetic resonance and density, could give clarity he stated.^33^ In this way the depleted status of the oil reservoirs both made possible the development and new geophysical insights, and positioned geophysical methods based on measuring radioactivity in a prime position to be embraced by the oil industry.

## 4 Measuring Radioactivity to Search for Oil

Here, in the literal residues of the expanding oil industry, the research to radioactive applied geophysics was developed. Both the geophysical science focusing on the properties of radioactivity, and early industrial applications emerged on sites such as Celle and Pechelbronn. These oil fields that had kickstarted the oil industry in its early days were now depleted, soiled, and in danger of being discarded in favor of more productive fields. Measuring radioactivity changed the purpose of these fields, however. By introducing methods to extend their productivity and fulfilling its new purpose as training ground for new geophysicists and geophysical technologies, the application of early quantum mechanics ignited a development that would lead to the large‐scale use of radioactive knowledge in the oil industry and the later entanglement between the oil and uranium industries in the decades following the Second World War.

This development had started with the applied research by physicists like John Joly during the first few years of the 20th century and was established as an independent field with the efforts of Richard Ambronn to bring together the discipline of applied geophysics. From there, the research and its possible applications were picked up by industrial oil parties scouting the oil field research for new methods to use in a growing, and competitive, oil sector. Schlumberger was one of the companies to take the lead in developing industrial applications. Already in 1910, at an early oil geology conference in Brussels, German physicists had claimed to have measured unusual high levels of radioactivity in rocks immediately below oil reservoirs in the Lower Saxony area, but Ambronn's publication really drew the attention of Conrad Schlumberger, one of the two founding brothers of the company, to the possibilities for finding oil that were offered by measuring radioactivity. From 1920s onwards Conrad Schlumberger steered the geophysical research of the company at Pechelbronn toward a systematic study of drilling mud to detect radioactivity, contracting several physicists that had previously worked independently as scientists working on radioactive measurements at Pechelbronn.^34^

At Pechelbronn, and at oil wells in the Soviet Union and Eastern Europe, most notably in Romania where high levels of radioactivity were measured in the local petroleum reservoirs, Schlumberger's geophysicists then worked on further developing the first radioactive well logging techniques.^35^ Numerous patents were registered, especially in the United States. There, the study of radioactivity phenomena in boreholes developed considerably since the first practical applications of this method were undertaken by various organizations around the late 1930's. On October 29, 1938, natural radioactivity was recorded for the first time in a well at Oklahoma City by a group of physicists of Engineering Laboratories in Tulsa, Oklahoma, among which Segre A. Scherbatskoy and Jacob Neufeld. With the support of Socony Vacuum (later Mobil Oil), the physicists established a service company, called Well Surveys, Inc., that would market the radioactivity log from 1939 onward.^36^

Practical applications were found soon. No matter the properties of the mud, the results from the radioactive well logging devices proved comparable with more traditional resistivity logs, and sometimes even better. Especially for the resumption of production in old wells, where no logging had ever taken place or where electric well logging devices had been useless, radioactive well logging ramped up the production rates. Unsurprisingly, the oil industry jumped on the development of this new geophysical device. In 1940, Schlumberger proposed new methods to use radioactive tracers to mark productive zones, and the Well Surveys, Inc. merged with the Lane Wells company to further develop geophysical technologies based on measuring radioactivity.^37^ Also other oil companies quickly recognized the potential of using radioactive well logging techniques to improve their production. Although wartime personnel and equipment shortages delayed the development of radioactive well logging devices in some parts of the oil sector, companies like Shell Oil entered the market by studies at their geophysical research laboratory in Houston, and the Texas Company combined their efforts with Schlumberger to develop a gamma‐ray logging patent.^38^

## 5 Fueling the Energy Transitions of the Twentieth Century

In the following decades, radioactive well logging devices became an essential tool in an oil industry rapidly evolving to supply an increasingly petroleum hungry world. Oil surpassed coal as primary energy source in a, mainly Western, world where growing welfare established an ever‐expanding demand. Measuring radioactivity offered an important asset for the oil geophysicists to find the needed new resources. Especially the neutron log, an offshoot of the traditional gamma‐ray logs, became an influential device. Already in 1938, geophysicists at Schlumberger were granted a patent for the adaptation of the neutron to lithological studies, and another patent was granted in 1940 to the Dutch physicist Folkert Brons.^39^ This research was then picked up in the United States where Well Surveys, Inc. undertook their first tests in 1941 which established that the highly penetrative character a flux of the neutron particles did augur well for logging through the casing.^40^ After the Second World War, companies like Schlumberger invested millions in programs to further develop this technique that would be used in various exploration parties in throughout the world.^41^

Besides offering tools to increase oil production, measuring radioactivity also proved valuable for finding another mineral that would become an essential resource for the late 20th Century energy mix: uranium. Already before the Second World War, when uranium was often discarded as a waste product in the search for the more valuable element radium, the radioactive mineral was regularly found in relation to petroleum reservoirs. Geophysicists at Schlumberger already reported in 1910 the findings of high amounts of radioactive minerals underneath oil deposits in the Lower Saxony region by German geologists, and later worked on Romanian oil sources with seemingly unusual high levels of radioactivity.^42^ Also in the United States, there were ample examples of entrepreneurs searching for either oil or radium and finding both petroleum and radioactive minerals.^43^

That these findings were no coincidence was established by several geophysicists within the context of the Manhattan District Project researching the appearance of uranium deposits in various sedimentary layers. To also supply the potential American nuclear industry with fissile materials after the Second World War, a committee was established, managed by former Shell geologist Paul L. Guarin, to document the various deposits all over the world.^44^ One of Guarin's assistants, the later professor in geophysics at Amherst College George Bain, studied the relation between uranium deposits and their regular appearance near amassed hydrocarbons. During the Second World War, and later for the American Atomic Energy Commission, Bain argued that uranium tends to group together around decaying biomass and in porous rocks. This often happened in petroleum source rocks, the same locations where crude oil would be produced before seeping away to deposit in vaults between sedimentary layers.^45^

As various historians of science have shown, this knowledge enabled geophysicists to make use of the devices created in the oil industry to search for uranium, and many oil entrepreneurs to join the various uranium booms in the decades following the Second World War.^46^ In this way, the devices built on the research conducted at the depleted oil wells of the Lower Saxony region and Pechelbronn fueled at least two of the important energy transitions of the late twentieth century. Both the rapidly growing oil sector and the emerging nuclear industry benefitted from the development of radioactive applied geophysics that took place in the repurposed oil fields which were endangered to become leftovers of the early oil industry.

## 6 Concluding Observations

Energy transitions are muddy phenomena. Focusing on the origin story of the intertwined history of the oil and nuclear industries helps to reframe some traditional concepts used in transition studies. First, the use of the boreholes at Lower Saxony, Ontario and Pechelbronn shows how residues of a growing industry became crucial hotspots for scientific and technological innovations that shaped the further exploitation of the oil industry. During the first decades of modern oil drilling, the wells were the epicenters of the rapidly developing oil industry, attracting many new companies and entrepreneurs. When water and mud took over however, the wells’ production rates started to decline, and geophysicists were invited to conduct their research on the rapidly devaluating oil infrastructures. There, they developed knowledge and technologies that would benefit the oil industry in their continuing search to fulfill the world's petroleum demand by finding new oil reservoirs, and sometimes extend the lifespan of the oil wells previously considered being depleted. By doing so, the residues of the early oil industry served as an incubator for the later transition of oil becoming the primary energy source.

At the same time, the geophysical research to new techniques to measure radioactivity also made possible the later diversification of various oil actors into nuclear energy. Technologies such as the radioactive well logging devices tied the oil industry to the worldwide uranium exploration projects that were conducted during and after the Second World War. In this way, the boreholes in these regions, with their specific geological qualities, birthed both the transition of oil to the primary energy source as well as the later development of nuclear energy during the second half of the 20th century.

This story marks the importance of combining the history of science and business history in understanding why transitions happen. Without considering the business reasons for industrial parties to allow geophysical research on their oil wells, and the industrial applications of applied geophysics that were deliberately promoted by geophysicists such as Richard Ambronn, one would not be able to fully appreciate the later interconnectedness of both the oil and uranium industries and would miss how this entanglement could become the birthplace of the 20th century energy mix.

Studying these early entanglements, shows us how later spillovers between seemingly competing, and very different, energy technologies are often based on characteristics that are inherent to resources needed for both industries. The quality of oil reservoirs that they become muddy when almost depleted combined with the fierce competition within the Western oil industry made possible the development of well logging devices on the depleted oil wells that, contrary to the more common electrical logging devices, were resistant to the muddy conditions. This in turn, steered the oil industry toward later diversification projects into nuclear energy. Unknown to the geophysicists during the first decades of the twentieth century, however, the formation of radioactive mineral deposits and petroleum reservoirs had played out over millennia underground. So when the applied geophysicists conducted their research on the oil wells that had been made available to them, they were geared toward finding high rates of radioactivity which in turn stimulated the research on using radioactivity measurements to locate sedimentary layers and in due course the development of radioactive well logging devices by the oil industry.

## Footnotes

1 Graig et al. 2025, Rinehart 2024.

2 About MGT, https://www.mgt‐geo.com/about‐us.html (accessed 11 March 2024); University Internships—Geology, Geosciences or Geophysics (Celle, Germany) 2024 opportunities, https://careers.bakerhughes.com/global/en/job/R110868/University‐Internships‐Geology‐Geosciences‐or‐Geophysics‐Celle‐Germany‐2024‐opportunities (accessed 11 March 2024); GeoEnergy Celle e.V.: Kempetenz in Erdöl, Erdgas, Erdwärme, https://www.geoenergy‐celle.de/ (accessed 11 March 2024).

3 German Oil Museum's collection, https://www.erdoelmuseum.de/unser‐museum/sammlung (accessed 11 March 2024); Egloff 1955, on 433; Telford et al. 1990, on 1–6.

4
Oreskes and Doel 2002; Porter 1995.

5
Anduaga 2016; Yates 2004, on 46–49; Bowker 1994.

6
Lucier 2009.

7
Bron 2024.

8
Turchetti and Roberts 2014; Cantoni 2017, on 4–5; Bron 2024.

9
Leung et al. 2014, on 316–326; Geels 2014, on 21–40; Strunz 2014, on 152.

10
Mody 2022; Bron 2024.

11
Egloff 1955, on 433.

12
Ambronn 1928, on 107–108.

13 Ibid.; Eve 1928, on 192.

14
Eve 1907, on v–vii.

15 Ibid., on 108; Rutherford and Soddy 1902.

16
Ambronn 1928, on 108–110.

17 Ibid., on 110–123.

18 Ibid., on 118–123; Egloff 1955, on 433; Allaud and Martin 1977, on 268.

19
Ambronn 1928, ibidem, on 108.

20
Joly 1909; Wyse Jackson 2006, on 227–231; Allaud and Martin 1977, on 268.

21 Ambronn 1977, on 125.

22
Mache and Bamberger 1914; Poole 1915.

23
Fletcher 1912.

24
Smeeth and Watson 1918; Raychaudhuri 2021, on 73–85.

25
Eve and McIntosh 1907; Burton 1904.

26
Mellor 2018, on 41–54; Bailes 1990, on 44–66.

27
Büchner 1912.

28
Graig et al. 2018; Rinehart 1930.

29
Anduaga 2016, on 22–23; Oreskes 2000, on 23–40; Priest 2014, on 42–44; Servos 1984, on 148; Yoder 2004, on 134–143; Forbes and O’Beirne 1957, on 60.

30
Bowker 1994, on 33–39.

31
Allaud and Martin 1977, on 268.

32 Sondage radioactif, étude de Clark Goodman du centre de Ridgefield sur des résultats expérimentaux (notes), 1951, Fonds Schlumberger, Fondation Musée Schlumberger, Crèvecoeur‐en‐Auge, AM‐69; Reinhardt and Gast 1995, on 704.

33
Ambronn 1928, on 7.

34 La radioactivité dans les sondages, étude de Louis Migaux sur les manifestations de la fluorescence et de la radioactivité (note de laboratoire), 1939, Fonds Schlumberger, Fondation Musée Schlumberger, Crèvecoeur‐en‐Auge, AM‐347; Carottage radioactive, instruction d'utilisation pour les sondages de gisements pétroliers (note technique), 1948, Fonds Schlumberger, Fondation Musée Schlumberger, Crèvecoeur‐en‐Auge, AM‐349; Bowker 1994, on 38–39, 117.

35 Carottage radioactive, instruction d'utilisation pour les sondages de gisements pétroliers (note technique), 1948, Fonds Schlumberger, Fondation Musée Schlumberger, Crèvecoeur‐en‐Auge, AM‐349.

36
Turchetti 2012, on 39–68; Allaud and Martin 1977, on 268.

37 Concurrence Lane‐Well, observation de leur procédé de carottage radioactif (patente, notes d’Eugène G. Leonardon), 1946–1950, Fonds Schlumberger, Fondation Musée Schlumberger, Crèvecoeur‐en‐Auge, AM‐556; Allaud and Martin 1977, on 268–270.

38 Radioactivité dans les sondages, expérience d'application de la Texas Company (rapport), 1942, Fonds Schlumberger, Fondation Musée Schlumberger, Crèvecoeur‐en‐Auge, AM‐835; Sondage radioactif, étude de Clark Goodman du centre de Ridgefield sur des résultats expérimentaux (notes), 1951, Fonds Schlumberger, Fondation Musée Schlumberger, Crèvecoeur‐en‐Auge, AM‐696; Allaud and Martin 1977, on 269–270; Forbes and O’Beirne 1957, on 206–207.

39
Fearon 1943; Allaud and Martin 1977, on 270–271.

40
Pontecorvo 1941; Turchetti 2012, on 39–68; Bonolis 2005, on 73; Bron 2024, on 42–43.

41 Sondage radioactif, instruction d'utilisation du logging à neutron par la SWSC (note technique), 1952, Fonds Schlumberger, Fondation Musée Schlumberger, Crèvecoeur‐en‐Auge, AC‐316; Allaud and Martin 1977, on 273.

42
Carottage radioactive, instruction d'utilisation pour les sondages de gisements pétroliers (note technique), 1948, Fonds Schlumberger, Fondation Musée Schlumberger, Crèvecoeur‐en‐Auge, AM‐349.

43
Rentetzi 2022, on 128–129; Bron 2024, on 37–40.

44
Parts of the Guarin geological report are reproduced in Brown and MacDonald 1977; Zoellner 2009, 48–49, 300; Helmreich 1986, on 44–45.

45 George Bain, Geology of Fissionable Materials, George W. Bain Papers, College Archives and Manuscript Collections, Amherst College, Box 10.

46
McLemore 1983, on 46; Amundson 2002, on 24–25; Cantoni 2017; Turchetti 2014; Carleson Ringholz 1989; Bron 2024, on 61–64.
